# Twelve month oral contraceptive pill prescriptions: Role of policy mandates on utilization

**DOI:** 10.1016/j.rcsop.2021.100094

**Published:** 2021-11-27

**Authors:** Samuel K. Peasah, Monal Kohli, Kiraat D. Munshi, Rochelle Henderson, Mark Mueller, Chronis Manolis, Yan Huang, Elizabeth C.S. Swart, Lynn Neilson, Chester B. Good

**Affiliations:** a Value-Based Pharmacy Initiatives, Center for High-Value Health Care, UPMC Health Plan, USA; b Health Services Research, Evernorth, USA; c Global Data & Analytics, Cigna, USA; d Pharmacy Services, UPMC Health Plan, USA

**Keywords:** Extended oral contraceptive pills, Mandates, Adherence, Discontinuation, Gaps, days' supply, Commercial health plans

## Abstract

Recent studies have suggested that extended duration oral contraceptive pills (OCP), such as the 12-month duration, have a positive impact on pregnancy rates but negative impact on pill wastage. Several states have since been mandating health plans to offer extended duration OCP as an option for women. The objective of the study was to evaluate the impact of these mandates on utilization of extended duration OCPs. Using claims data of a large pharmacy benefit manager for commercially insured women from 2018 to 2019, use, adherence, continuity, and wastage of OCPs by women dispensed one-month only, three-months only, 6 or 12-months only, and other months (which includes other months and mixed duration OCP) was retrospectively analyzed. OCP dispensed by year, and adherence, continuity, wastage over a 15-month period were summarized using Chi square and ANOVA. There were 874,420 and 875,914 women in this study in 2018 and 2019 respectively. Of these, 34% were from states with the mandate (SWM). Most women filled the one-month and three-month duration, with very low overall 6 or 12-month duration claims. Proportion of utilizers of 6 or 12- month duration was higher in SWM than in those without, although differences in absolute rates were very low. Patients with OCP discontinuation, gaps ≥7 and 14 days, were fewer among those filling 6 or 12-month duration but conversely, wastage was higher in this group compared to those filling one or three-month duration. Our findings suggest that, among commercially insured women, extended duration OCP mandates have so far not had much influence on use of 6 or 12-month duration OCP prescriptions.

## 1 Introduction

The U.S. Select Practice Recommendations (U.S. SPR), under the Centers for Disease Control and Prevention (CDC), included in its 2016 practice recommendations the provision and prescription of multi-pack oral contraceptive pills (OCP) up to one-year supply.^1^^,^^2^ These recommendations followed reports that prescribed or dispensed oral contraceptive multi-packs improve pill continuity, reduce pregnancy tests requests and pregnancies.^3^, ^4^, ^5^ Subsequently, some researchers and advocacy groups have advanced the need for insurance policies that allow for prescribing and dispensing of OCP multi-packs beyond three-month duration by third-party payers and dispensers.^6^^,^^7^ As of 2019, there were 17 states and Washington DC with policies mandating health plans to provide at least six-month duration dispensing as an option for women.^8^

OCPs are available in either single or multi-packs. They are broadly grouped as combined (consisting of estrogens and progestin either as a 21-day or 28-day single-packs), progestin only (single-packs), extended [such as levonorgestrel/ethinyl estradiol, 91-day packs with 84 active pills], or continuous [such as levonorgestrel/ethinyl estradiol with 365 active pills].^8^ Third-party payers often use quantity limits for patient safety and cost containment reasons. They most often allow and encourage multi-month prescriptions.^9^ However, there are concerns for many insurers that extended duration packs (6-month or 12-month duration OCP prescriptions) might also be associated with medication wastage due to lost supplies, change in medication, or change in one's reproductive goals. Three-month duration has become a popular choice for both retail and mail-order pharmacies to improve adherence, combined with interventions such as automatic refills.^10^

Adherence to OCPs measured as proportion of days covered (PDC) was 68% and by self-report, 76% according to a recent study.^11^ Non-adherence to oral contraceptives can have significant consequences for individuals and for the healthcare system. A recent study from California reported that a 12-month duration OCP improved medication adherence (i.e., reducing the gaps in refills) and therefore reduced negative outcomes (such as unplanned pregnancy).^5^ Counterbalancing the report of the California study are concerns of medication wastage with provision of OCPs beyond three-month duration.

In this study, OCP users in states that have mandated extended duration packs as an option was examined, and the association between number of days' supply dispensed (extended duration vs. three-month, extended duration vs. one-month, extended duration vs. other) and OCP use continuity (medication adherence, gaps, discontinuation) or wastage was evaluated. The primary objective of this study is to assess the role of the extended duration pack policy on utilization. To our knowledge this is the first report on the role of state policy on extended duration OCP utilization uptake and thus provides insight to pharmacists, prescribers, and health plan payers on OCP extended duration usage, wastage, and continuity across the United States (US).

## 2 Methods

This study used insurance eligibility and prescription claims data of commercially insured members whose pharmacy benefits were managed by a large pharmacy benefit manager in the US that provides prescription drug benefits to more than 90 million individuals. Data were obtained for OCP prescriptions from January 1, 2018, to December 31, 2019. Inclusion was limited to women who (a) were continuously eligible with pharmacy benefits from January 1, 2018, until December 31, 2019, (b) were aged 15 to 49 years old as of January 1, 2018, and (c) had at least one OCP claim. Those with only emergency contraceptive claims (e.g., Plan B One-Step) were excluded from the study. Those in the final sample were assigned to one of four groups based on the days' supply of OCP filled throughout the two-year study period. These groups consisted of, (1) one-month duration (days' supply of 21–30 days); (2) three-month duration (days' supply of 63 or 83–91 days); (3) 6 or 12-month duration; and (4) other-month duration (including mixed-month duration). Those filling three-month supply of the 21 pills-pack of oral contraceptives (63 days' supply) were included in the three-month duration group.

Seventeen states and Washington DC have passed legislature to mandate 6 or 12-month duration OCP dispensing as an option for patients. The states, year the laws were passed and implemented, are displayed in Table 1 including the states in this study. This retrospective, observational study was divided into two parts.

**Table 1:** States and when Contraceptive Laws were Enacted and Implemented.

Year law passed	States	Date implemented
2015	Washington DC	01/01/2017
2016	California*	01/01/2017
	Hawaii*	01/01/2017
	Illinois*	01/01/2017
	Oregon*	01/01/2016
	Vermont	10/01/2017
2017	Maine**	01/01/2019
	Massachusetts*	08/2018
	Nevada*	01/01/2018
	New Jersey	03/2018
	New York*	08/22/2017
	Virginia**	01/01/2018
	Washington*	01/01/2018
2018	Connecticut*	01/01/2019
	Delaware*	07/11/2018
	Maryland	01/01/2020
	New Hampshire**	01/01/2019
	Rhode Island	04/01/2019
2019	Colorado*	07/01/2019
	New Mexico	01/01/2020

States in the 2018 study sample*.

States in the 2019 study sample**.

### 2.1 Cross-sectional study design

This part was a retrospective, cross-sectional design examining utilizers of OCP in 2018 and 2019 for women residing in states with an extended duration policy mandate (SWM) and women residing in states without such a mandate (SWOM).

### 2.2 Cohort study design

This part of the study examined medication adherence, gaps in OCP use, discontinuation, and wastage of OCP through a retrospective cohort study design. Women with ≥1 claim for OCP between January 1, 2018, and September 30, 2018 (index period) were included in this cohort analysis, with the first OCP claim in the index period designated as the index claim. They were each followed for 15 months for the measures such as adherence, discontinuation, and wastage. The most used duration for adherence measures is 12-months but durations below and above 12-months, including 6-months, 9-months, 15-months, 18-months, and 24-months have been used.^12^, ^13^, ^14^ We used 15-months to allow patients with extended packs to have at least one opportunity of not refilling their medications. We recognize that it gives undue advantage of better adherence to longer day supply packs over shorter duration packs. All contraceptive claims, including non-oral, were used for some outcome measurements. Data used for this study were de-identified and in full compliance with the Health Insurance Portability and Accountability Act (HIPAA); therefore, an institutional review board approval was not required.

### 2.3 Outcomes of interest

The following outcomes were examined in this study:

1) Utilizers: The first part of the analysis (cross-sectional) examined the number and proportion of women filling at least one OCP prescription grouped by days' supply of filled claims
2) Medication adherence: Measured as PDC, adherence was estimated as the proportion of days in a 15-month period post-index claim during which study participants had active OCP prescriptions. PDC was estimated as a percentage with 100% indicating perfect adherence.
3) Gaps: The number and proportion of participants with a gap in subsequent fill of OCP claim of ≥7 days and ≥14 days were calculated. Gaps were calculated as the number of days elapsed before the subsequent fill of oral contraceptive.
4) Discontinuation: Medication discontinuation was estimated in two ways: a) Discontinuation by days, which was calculated as gap of ≥30 days before stoppage or subsequent fill of OCP; and b) Discontinuation by method, which was calculated as a switch from oral contraceptive to another method of contraception (not involving oral drugs).
5) Pill wastage: Pill wastage was estimated in two ways: a) Pill wastage by drug switch was defined as switching to another oral contraceptive with at least two cycles of the previous oral contraceptive drug remaining; and b) Pill wastage by method switch was defined as switching from oral contraceptive to another method of contraception (such as intrauterine devices) with at least two cycles of the oral contraceptive drug remaining. Pill wastage was estimated for those in the three-month, and extended duration groups.

### 2.4 Statistical analysis

Comparison of OCP utilization rates between patients in SWM and SWOM among the 4 groups (only one-month, only three months, only 6 or 12 months, and other duration) was analyzed using one-way analysis of variance (ANOVA). Medication adherence, measured as average PDC, was also analyzed for the four patient groups using ANOVA. Chi-square test of proportions was used to examine the proportion of participants with medication gaps ≥7 days and ≥14 days as well as the proportion of those discontinuing their oral contraceptive regimen across the four patient groups. Statistically significant differences were accepted at *p* < .05. SAS 9.4 software (SAS Institute Inc., Cary, NC, USA) was used for all statistical analyses.

## 3 Results

The total number of women with OCP claims, excluding those with only emergency contraceptive claims, were 874,420 and 875,914 in 2018 and 2019 respectively. Of these, 298,216 (34%) were from SWM in 2018 and similar number in 2019. There were 11 and 14 SWM in our cohort with the extended duration supply mandate in 2018 and 2019 respectively. Most of the 202 participants with only extended duration supply in 2018 were from Washington (54%), New York (16%), Hawaii (4.5%), Oregon (4.5%) and Texas (4.5%). Similarly, in 2019, of the 369 with extended duration packs, 51% were from Washington, 18% from New York, 5% from Massachusetts, 4% from California and 4% from Oregon. The total number of participants in the cohort, followed for 15 months, for the adherence, discontinuation, wastage, and gaps analyses was 816,828.

In Table 2, the most common duration of OCP prescriptions filled based on days' supply across all age groups in 2018 was one-month but three-month duration was more popular in 2019 for women residing in SWM. Although, compared to SWOM, SWM had higher proportion of extended duration supply (2018: 0.06% vs. 0.004%, 2019: 0.11% vs. 0.006%), the proportions were significantly lower than one-month (2018: 43% vs. 47%, 2019: 36% vs. 43%), and three-month duration (2018: 31% vs. 32%, 2019: 40% vs. 37%). All the statistically significant findings were at *p* < .0001. The younger group of women residing in SWM (15–24 years) had higher utilization rates of the 6 or 12-month duration than the older group (35–49 years).

**Table 2:** Utilization of OCP by multi-pack mandate, number of packs dispensed, and age group in 2018.

	Had ≥ six-month mandate in effect	Women with an index OCP claim	Only one- month supply	Only three- month supply	Only 6 or 12-month supply	Other OCP supply durations
Age 15–19	Yes	51,596	24,372 (47%)	12,594 (24%)	33 (<1%)	14,597 (29%)
	No	103,258	53,036 (51%)	26,929 (26%)	~ (<1%)	23,290 (23%)
Age 20–24	Yes	76,979	31,626 (41%)	22,887 (30%)	61 (<1%)	22,405 (29%)
	No	148,517	69,545 (47%)	46,059 (31%)	~ (<1%)	32,902 (22%)
Age 25–29	Yes	40,903	17,555 (43%)	11,946 (29%)	24 (<1%)	11,378 (28%)
	No	75,979	37,687 (50%)	23,184 (31%)	~ (<1%)	15,106 (20%)
Age 30–34	Yes	44,573	20,104 (45%)	13,849 (31%)	33 (<1%)	10,587 (24%)
	No	81,638	40,566 (50%)	25,980 (32%)	~ (<1%)	15,087 (18%)
Age 35–39	Yes	36,595	15,869 (43%)	12,365 (34%)	15 (<1%)	8346 (23%)
	No	70,968	33,370 (47%)	24,558 (35%)	~ (<1%)	13,040 (18%)
Age 40–49	Yes	47,570	17,598 (37%)	19,245 (40%)	11 (<1%)	10,716 (23%)
	No	95,844	38,008 (40%)	39,679 (41%)	~ (<1%)	18,153 (19%)
All Ages 11–49	Yes	298,216	127,124 (43%)	92,886 (31%)	177 (<1%)	78,029 (26%)
	No	576,204	272,212 (47%)	186,389 (32%)	25 (<1%)	117,578 (20%)

All differences were statistically significant at P < .0001.

OCP: oral contraceptive pills.

~ less than 20 people.

**Fig. 1:**
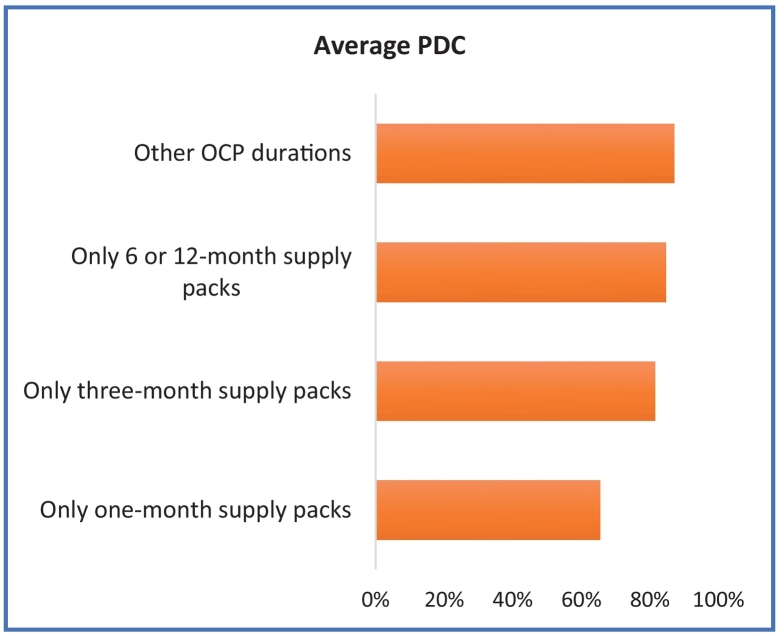
Proportion of Days Covered (PDC) by Number of Packs Dispensed.

**Fig. 2:**
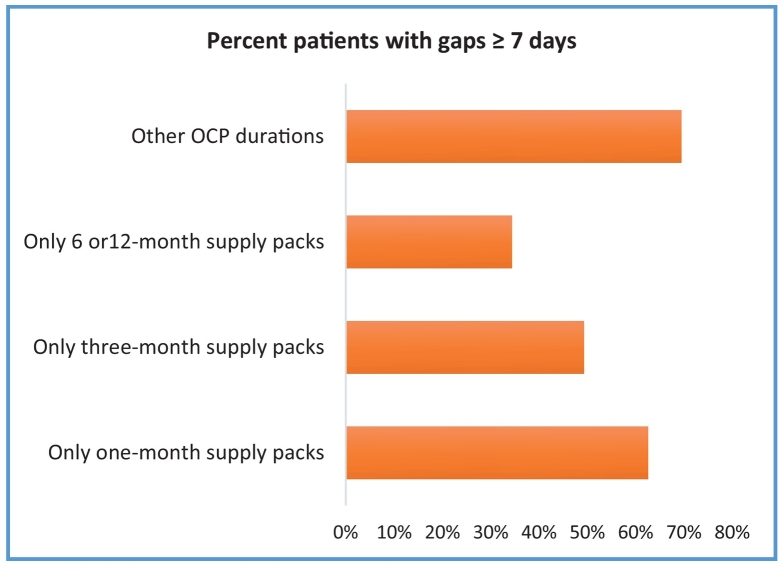
Proportion of Participants with Gaps ≥7 days in Refills by Number of Packs Dispensed.

**Fig. 3:**
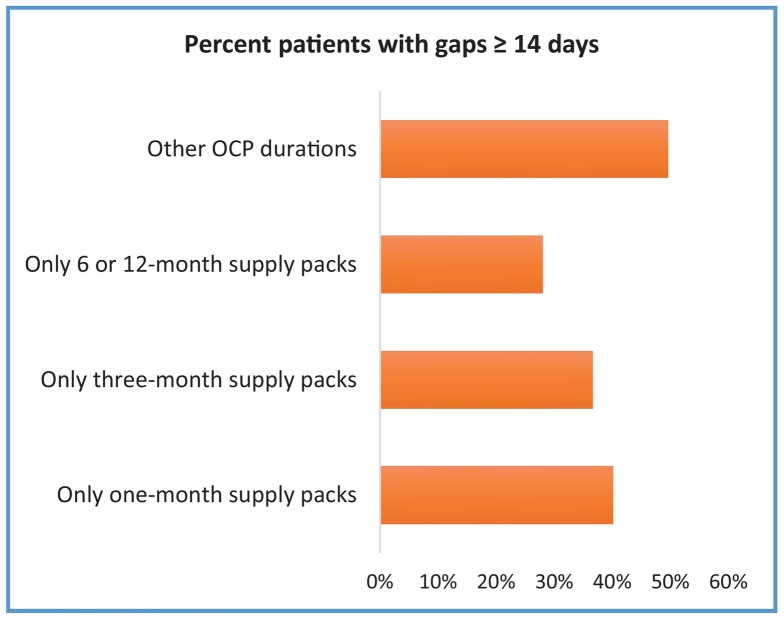
Proportion of Participants with Gaps ≥14 days in Refills by Number of Packs Dispensed.

In Table 3, in the index period, OCP usage of most common duration in both groups was one-month followed by three-month duration. Adherence was lowest for the one-month duration packs dispensed (Fig. 1). Conversely, women with extended duration packs had the lowest ≥7 and ≥ 14-day gaps without refills (Fig. 2, Fig. 3).

**Table 3:** Discontinuation of OCP by number of packs dispensed and type of discontinuation.

	Women with an index OCP claim N (%)	Discontinuation by Method N (%)	Discontinuation by Days' Supply N (%)
Only one-month supply packs	359,708 (44%)	11,071 (3%)	88,695 (25%)
Only three-month supply packs	264,205 (32%)	4966 (2%)	55,291 (21%)
Only 6 or 12-month supply packs	157 (<1%)	~ (3%)	~ (13%)
Other OCP duration packs	192,758 (24%)	3258 (2%)	53,573 (28%)

All group differences were statistically significant at P < .0001.

OCP: oral contraceptive pills.

~ Less than 20 people.

Table 3 also summarizes discontinuation defined as either change in method of contraception from OCP to a different method, for example injectables- or change from an OCP to a different brand of OCP. Generally, discontinuation defined by days' supply was higher (12%–28%) than discontinuation defined by change in method of contraception (2%–3%). Women using only one-month or extended duration supply generally discontinued at a higher rate when examining discontinuation by method (3.1 and 3.2% respectively), while those with extended duration had the lowest discontinuation rates when measured as days' supply (12.7%).

Of the 264,205 women who only took the three-month supply, 2% switched to a different OCP brand resulting in an average of 69 days' supply not used (wasted). Similarly, the proportion that switched to another contraceptive method (0.4%) also wasted 70 days' supply of the OCP (Table 3). There was no difference between rates in SWM and SWOM. Conversely, of the 157 women who only received the extended duration, 5.1% switched to a different OCP brand resulting in an average of 232 days' supply wasted and 1.3% switched to another contraceptive method for 225 days' supply wasted, with almost all occurring in SWM (Table 4).

**Table 4:** OCP Pill-Wastage by number of packs dispensed and method of switching.

	Women with an index OCP claim N	Switch to generic alternative N (%)	Generic switch- Average days' supply wasted (Mean)	Switch to alternative method N (%)	Method switch- Average days' supply wasted (Mean)
Only three-month supply packs	264,205	5745 (2%)	1.6 (69 for switchers)	963 (<1%)	0.3 (70 for switchers)
Only 6 or 12-month supply packs	157	~ (8%)	12 (232 for switchers)	~ (2.0%)	3.0 (225 for switchers)

All differences were statistically significant at P < .0001.

OCP: Oral contraceptive pills.

~ Less than 20 people.

## 4 Discussion

### 4.1 Principal findings

The primary objective of this study was to describe OCP prescription patterns between states with and without policies mandating provision of multi-pack OCP prescription and dispensing. One-and three-month durations were most frequently prescribed to women whether in SWM or SWOM. Although there were higher utilization rates of extended duration packs in SWM compared to SWOM, the low rates suggest that the policy has not yet had any meaningful impact on extended duration usage among commercial insurers. While only 11 and 14 states had the extended duration mandates in 2018 and 2019 respectively, most of the utilization (71%–78%) came from only one state, Washington state, suggesting generally low utilization in SWM. Only Massachusetts and Oregon had at least 5% utilization.

There are additional contraceptive laws in a few states including Washington. Pharmacist-allowed OCP prescribing, no copays for both over the counter and prescription OCPs, no coverage or choice of method limits such as denials, step therapy or prior authorization for OCP, all may have contributed to the higher uptake of the extended duration in Washington.^15^ Some of the SWM require initial dispensing of three-month duration before a longer duration extended multi-pack is dispensed. Additionally, differences exist between when these OCP laws were passed, and when health plans were required to comply. For example, in states such as Washington, Nevada, Virginia, the law was passed in 2017 and health plans were required to comply on January 1, 2018. However, in some states such as Colorado and Maine, the OCP law was passed in 2017 but implemented in 2019.^16^ Some states like Massachusetts enacted the law in 2017 but implemented in August 2018. California is a unique situation; although the law was passed in 2016 and implemented on January 1, 2017, the state Medicaid Family Planning Expansion and Family PACT have allowed extended duration multi-pack supply of OCPs for over 25 years when dispensed at a clinic, which could explain the low utilization of OCP in California in our commercial cohort (if not billed through insurance). States such as Oregon and Virginia also allow extended duration multi-packs at clinics for Medicaid beneficiaries.^16^

Measuring contraceptive adherence is challenging as participants' medication adherence are often informed by need. A woman might decide to stop taking an OCP initially to become pregnant but rescind the decision soon after, creating a refill gap leading to lower adherence rate.^11^^,^^17^ Additionally, adherence/discontinuation may be influenced by changes in relationship status. However, researchers have used both claims' data and surveys to estimate OCP adherence rates. There was 30% discontinuation rate among women in the Highly Effective Reversible Contraceptive Initiative Salt Lake study.^18^ This rate was comparable to this study's findings of 17%–36% depending on number of days' supply dispensed. In another study comparing participants using seven-month duration and three-month duration, higher duration pack participants had a better continuation rate (51% vs. 35%).^5^ This rate is lower than the OCP continuation rates in this study. Their study was a randomized controlled study among women in an urban family-planning clinic compared to our observational study of commercially insured participants who filled their OCP through pharmacies.^5^

This study's findings also align with Foster et al.^4^ where younger women were more likely to receive 12-month duration packs than older women and 12-month duration multi-packs had higher continuation rates than three-month duration packs. This study's findings of wastage in extended duration packs also align with White et al.^5^ Like Borrero et al., women on three-month duration packs had fewer ≥7 days gaps compared to those on one-month duration, although this study's rates were lower (49% vs. 63%) compared to Borrero's (63% vs. 72%). Borrero's study was based on a Veterans Affairs (VA) patient population compared to commercially insured cohort.^19^

This study adds to the conversation regarding the decision of individual states, as well as individual health insurers, that are considering the merits of 6 or 12-month supply OCP multi-packs. First, it is apparent that as of 2019, state laws have had only a nominal effect, at best, on moving women from one-month or three-month duration OCP prescriptions to 6 or 12-month duration. Thus, concerns for wastage, at least at current 6 or 12-month duration utilization rates, should be allayed. Moreover, Judge-Golden et al.'s economic decision model suggest a favorable economic outcome if 12-month duration dispensing is used compared to 3-months.^6^ In that study, the authors modelled 24,309 women in reproductive age within the VA system. Relying on improved continuity of use and reduced unintended pregnancies of 12-month extended duration packs, the model suggested $87 per woman per year savings for the VA health system. Second, the substantial use of one-month duration prescriptions was surprising and suggests an opportunity to improve medication compliance by transitioning many of those women to three-month or longer duration OCP prescriptions.

### 4.2 Strengths and limitations

A key strength of our study is the availability of data from almost all states with the mandate. However, since this study is an observational study, it is subject to non-randomized control trial biases such as selection bias. Additionally, it is assumed that all participants were taking the OCP to prevent pregnancy and emergency contraceptive usage was excluded. Currently, 22% of women use OCPs, of which 86% use it to prevent pregnancy.^8^^,^^20^ Although PDC is a widely used measure of adherence, it is still based upon prescription claims and not actual, verifiable, pill usage. Secondly, patients on extended packs or higher duration packs have a lower opportunity to default in refilling their OCP. It is impossible to know whether participants or prescribers were aware that 6 or 12-month duration OCP multi-packs were available in states with policy mandates. This study was based on commercially insured women and therefore does not represent the usage patterns of women who use free-clinics and family-planning clinics for OCPs instead of community pharmacies. Finally, the impact of state policies on unplanned pregnancies and abortions were not examined.

This study's findings suggest that among women who have commercial insurance, the 6 or 12-month OCP mandate has not significantly influenced utilization, and very few women take advantage of that option. It also supports the literature that 6 or 12-month duration have better adherence/continuity rates but higher pill wastage. One-month duration utilization rate is very high overall regardless of state mandates for extended OCP supplies, therefore encouraging prescribers to switch women to at least a three-month supply pack (where adherence is higher than one-month supply packs) could be beneficial to most women.

Community pharmacists can also play an important role of identifying patients who will benefit from 6 or 12-month extended duration packs as part of their medication management practices. Future studies, especially in family planning clinics or community pharmacies, could examine the impact of 6 or 12-month duration OCP state mandates on unplanned pregnancies and abortions.

## Funding

This work was funded by Evernorth.

## Declaration of competing interest

The authors are/were employees of either UPMC Health Plan or Evernorth/Cigna during the project. This project is part of an ongoing collaborative partnership between the two organizations and hence we do not report of any conflict of interest.
